# Employment and insurance outcomes and factors associated with employment among long-term thyroid cancer survivors: a population-based study from the PROFILES registry

**DOI:** 10.1007/s11136-015-1135-z

**Published:** 2015-09-22

**Authors:** S. J. Tamminga, U. Bültmann, O. Husson, J. L. P. Kuijpens, M. H. W. Frings-Dresen, A. G. E. M. de Boer

**Affiliations:** Coronel Institute of Occupational Health, Academic Medical Centre, University of Amsterdam, Amsterdam, The Netherlands; Department of Health Sciences, Community and Occupational Medicine, University Medical Center Groningen, University of Groningen, Groningen, The Netherlands; Department of Medical Psychology, Radboud University Medical Center, Nijmegen, The Netherlands; Comprehensive Cancer Center Netherlands South, Eindhoven Cancer Registry, Eindhoven, The Netherlands; VGZ Health Insurance Company, Eindhoven, The Netherlands

**Keywords:** Thyroid cancer, Cancer survivorship, Work, Employment, Population-based study

## Abstract

**Purpose:**

To obtain insight into employment and insurance outcomes of thyroid cancer survivors and to examine the association between not having employment and other factors including quality of life.

**Methods:**

In this cross-sectional population-based study, long-term thyroid cancer survivors from the Netherlands participated. Clinical data were collected from the cancer registry. Information on employment, insurance, socio-demographic characteristics, long-term side effects, and quality of life was collected with questionnaires.

**Results:**

Of the 223 cancer survivors (response rate 87 %), 71 % were employed. Of the cancer survivors who tried to obtain insurance, 6 % reported problems with obtaining health care insurance, 62 % with life insurance, and 16 % with a mortgage. In a multivariate logistic regression analysis, higher age (OR 1.07, CI 1.02–1.11), higher level of fatigue (OR 1.07, CI 1.01–1.14), and lower educational level (OR 3.22, CI 1.46–7.09) were associated with not having employment. Employment was associated with higher quality of life.

**Conclusions:**

Many thyroid cancer survivors face problems when obtaining a life insurance, and older, fatigued, and lower educated thyroid cancer survivors may be at risk for not having employment.

## Introduction

Cancer is no longer considered a death sentence as many of the patients diagnosed with cancer survive due to improved screening, diagnosis, and cancer treatment [1]. This particularly applies for differentiated (papillary and follicular) thyroid cancer survivors, as the 5-year survival rate exceeds 90 % [2] and the 10-year survival rate exceeds 70 %. This high survival rate implicates that survivorship care becomes highly relevant and important for this patient group [3].

For all cancer survivors within the working age, one aspect of survivorship includes the ability to remain in or return to work. Unfortunately, it is known from other cancer types that many cancer survivors experience unwanted changes in their employment status such as working part-time due to cancer [4] and financial status [5] or experience problems upon their return to work [6, 7]. These adverse work outcomes arise due to various factors such as long-term fatigue, unsupportive work environment, and physically heavy work [8, 9].

It is unfortunate that cancer survivors experience such adverse work outcomes because return to work is considered a key aspect of survivorship. Most cancer survivors attribute great meaning to work: it contributes to social inclusion [6], higher self-esteem [10], a better financial situation [5], and it contributes to better quality of life [11]. For those reasons, it is important to prevent adverse work outcomes for all cancer survivors.

Besides adverse work outcomes, it is known from other cancer types that cancer survivors experience socio-economic consequences such as problems with obtaining life and health care insurance [12] or an increase in life or health care insurance premiums [13]. These insurance problems may have a negative impact on cancer survivors’ financial situation and quality of life [13]. Therefore, it is important to draw attention not only to the adverse work outcomes of cancer survivors but also to these other socio-economic consequences of a cancer diagnosis.

Studies among thyroid cancer survivorship including employment and insurance issues are scarce [14], since most work-related survivorship studies focused primarily on breast cancer survivors [8]. However, it is very relevant to study the work and insurance outcomes of thyroid cancer survivors because they are relatively young compared to other cancer survivors and are therefore more often of working age and in a stage of life in which they want to obtain a life insurance and mortgage. Furthermore, they have a high chance of survival, but suffer from higher levels of fatigue compared to the general population [15], which might limit employment possibilities and obtaining the necessary insurances.

Therefore, the aims of the present study are: (1) to examine the consequences of a thyroid cancer diagnosis on work and insurance outcomes, (2) to study which factors are associated with these work outcomes, and 3) to study thyroid cancer survivors with and without paid employment on quality of life outcomes.

## Methods

### Setting and participants

This study was a cross-sectional population-based study from the southern parts of the Netherlands. The methods of this study have been described in detail elsewhere [15, 16]. Cancer survivors diagnosed with papillary, follicular, or medullary thyroid cancer between 1990 and 2008 and registered in the Eindhoven Cancer Registry (ECR) were eligible for participation. Patients with anaplastic thyroid cancer were excluded because of the very poor prognosis of this type of cancer. Cancer survivors were further excluded for this current study if they: (1) were too ill to participate (*n* = 31), had unverifiable addresses (*n* = 70), died prior to the study (*n* = 6), were not allowed to be contacted as decided by their hospital (*n* = 86), or were not aged 18–65 years (*n* = 118). This resulted in a final study population of 257 cancer survivors. The certified Medical Ethical Committee of the Maxima Medical Centre in Veldhoven judged that ethical approval was not required for this study.

### Data collection

Data collection was performed within PROFILES (Patient Reported Outcomes Following Initial treatment and Long term Evaluation of Survivorship) [16] and started in November 2010. Eligible cancer survivors were informed about the study via a letter from their (former) treating physician, including an informational leaflet containing a link to a secured website, a password, and a login. Cancer survivors who were willing to participate could provide informed consent and fill in the questionnaire via the secured website or by a paper version.

### Measures

Two types of measures were used. Clinical and socio-demographic characteristics available through the ECR were linked with self-reported questionnaire data. Characteristics collected by means of the ECR included age at diagnosis, gender, type of thyroid cancer (papillary, follicular, or medullary), stage at diagnosis according to the tumour–node–metastasis (TNM) clinical classification [17], and primary cancer treatment. The questionnaire included the following work-related characteristics: employment status, reason for not being employed, number and type of work changes due to cancer, actual number of hours working, financial difficulties due to cancer, and a question to assess whether patients were concerned about not being able to work if they would become ill again. The questionnaire further included questions on problems with obtaining health care insurance, life insurance, and mortgage and questions on comorbidity, marital status, years since diagnosis, age at time of survey, educational level, depression, anxiety, thyroid-specific health-related quality of life, overall quality of life, global health, and fatigue.

Financial difficulties were measured with a single item from the EORTC health-related quality of life questionnaire and dichotomised into ‘not at all’ and ‘a little’ and compared to ‘quite a bit’ and ‘very much’ [18]. Comorbidity was assessed with the self-administered Comorbidity Questionnaire, containing a list of 14 comorbidities (present/not present), and patients were asked whether they perceived each comorbidity as a hindrance in their daily activities at the time of the survey (yes/no) [19].

Anxiety and depression were assessed with the Hospital Anxiety and Depression Scale (HADS) [20]. The scale contains 14 items consisting of HADS-A (anxiety, 7 questions) and HADS-D (depression, 7 questions) subscales [21]. All items are rated on a four-point scale (0–3) with higher scores indicating higher levels of depression and anxiety. Thyroid-specific health-related quality of life was assessed with the reliable and valid THYCA-QoL [21]. The THYCA-QoL consists of 24 items (1–4) with lower scores indicating less symptoms and higher quality of life and has seven scales: neuromuscular, voice, concentration, sympathetic, throat/mouth, psychological, and sensory problems. Fatigue was assessed with the validated and reliable Fatigue Assessment Scale (FAS) for use among cancer survivors [22], which contains 10 items which are scored on a 5-point scale with higher scores indicating more fatigue. Global health perception was measured with a single item of the Short Form-12 [23] ranging from ‘poor health’ to ‘excellent health’. Overall quality of life was measured on a 7-point Likert scale [18], with higher scores indicating a better quality of life.

### Statistical analysis

Statistical analyses were done using SPSS version 20.0. We considered a *p* value of ≤0.05 statistically significant. Socio-demographic, clinical, employment, and insurance outcomes were reported using descriptive statistics.

Univariate logistic regression analyses with age, gender, educational level (high (reference) versus medium/low), marital status (married or living with partner (reference) versus no partner), cancer diagnosis (papillary (reference) versus follicular), cancer treatment (surgery only (reference) versus surgery and additional treatment), cancer stage [1 and 2 (reference) versus 3 and 4], fatigue, anxiety, depression, and comorbidity (no comorbidity (reference) versus one or more comorbidities) were conducted to identify associations with not having employment. We choose these factors as these were found to be associated with employment among other groups of cancer survivors [8, 9]. Factors that were statistically significant were entered in a multivariate logistic regression analysis unless the correlation coefficient between two variables was ≥0.7 to prevent multicollinearity. The model was built in three blocks: (1) socio-demographic variables, (2) clinical variables, (3) long-term side effects variables, and (4) demographic, clinical, and long-term side effects variables. Odd ratios (ORs) will be reported with 95 % confidence intervals (CI).

Differences between thyroid cancer survivors with and without paid employment on the thyroid-specific health-related quality of life scales, global health, and quality of life were analysed using Student’s *t* test when variables are normally distributed or the Mann–Whitney *U* test otherwise. To test whether variables were normally distributed, we used Kolmogorov–Smirnov test of normality (cut-off *p* value ≤0.05).

## Results

Of the 257 cancer survivors, 223 returned the questionnaire (response rate 87 %). Table 1 shows the sample characteristics. The mean age was 49.5 (standard deviation ± 9.8) years, and 22 % were male. Almost three-quarter (71 %) of the patients were diagnosed with stage 1 disease. Surgery followed by ^131^I therapy (70 %) was the most common treatment. The median time since diagnosis was 9.0 years.

**Table 1:** Sample characteristics of thyroid cancer survivors

Socio-demographic characteristics		
Age in years at time of study (mean ± SD)	49.5 ± 9.8	*N* = 223
Gender [*N* (%) male]	49 (22)	*N* = 223
Marital status [*N* (%) married or living with partner]	187 (84)	*N* = 223
Educational level [*N* (%)]		
High	68 (31)	*N* = 222
Intermediate	108 (49)	
Low	46 (21)	
Clinical characteristics		
Years since initial diagnosis (mean ± SD)	9.9 ± 5.2	*N* = 223
Type of thyroid cancer [*N* (%)]		
Papillary	171 (77)	*N* = 213
Follicular	41 (19)	
Medullary	9 (4)	
Primary treatment [*N* (%)]		
Surgery	63 (29)	*N* = 223
Surgery + ^131^I ablation	145 (70)	
Other	2 (1)	
Stage at diagnosis [*N* (%)]		
1	158 (72)	*N* = 220
2	26 (12)	
3	29 (13)	
4	7 (3)	
Number of comorbidity at time of study [median (range)]	0 (0–9)	*N* = 223
Fatigue (FAS) (mean ± SD)	21.8 ± 7.6	*N* = 212
Anxiety (HADS) (mean ± SD)	4.7 ± 3.9	*N* = 215
Depression (HADS) (mean ± SD)	3.2 ± 3.0	*N* = 215

*SD* standard deviation. Education: high (pre-university education, high vocational education, university), intermediate (lower general secondary education or vocational education), low (no or primary school). Stage: tumour–node–metastasis clinical classification. *FAS* Fatigue Assessment Scale. *HADS* Hospital Anxiety and Depression Scale. Higher score means higher fatigue, anxiety, and depression. Numbers do not always add up to 223 due to missing values. Percentages do not always add up due to rounding

### Employment outcomes and work changes

Seventy-one per cent of the thyroid cancer survivors were employed (Table 2). Reasons for not being employed were disability (33 %), early retirement (e.g. due to reconstitution) (14 %), no job (6 %), or other (e.g. voluntary unemployed) (46 %). One-third (33 %) reported work changes due to cancer, i.e. working less hours (16 %), being disabled (9 %), being fired (5 %), stopped working (4 %), re-educated (3 %), early pension (1 %), or working more hours (1 %) as work change due to cancer.

**Table 2:** Employment outcomes among thyroid cancer survivors

Employment outcomes		
Employment at this moment [*N* (%) employed]	154 (71)	*N* = 217
Reason for no employment [*N* (%)]		
No job	4 (6)	*N* = 63
Work disabled	21 (33)	
(Early) retirement	9 (14)	
Other	29 (46)	
Disability percentage [median (range)]	100 (30–100)	*N* = 19
Change in employment status due to cancer [*N* (%) yes]	74 (33)	*N* = 223
Type of work changes due to cancer [*N* (%)]^a^		
Fired	10 (5)	*N* = 87
Disability	21 (9)	
Early pension	2 (1)	
Stopped working	9 (4)	
Re-educated	6 (3)	
Working more hours	3 (1)	
Working less hours	36 (16)	
If employed, number of hours working (mean ± SD)	28 ± 12	*N* = 156
Financial difficulties due to cancer [*N* (%) yes]	21 (10)	*N* = 219
Concerned about not being able to work if become ill again		
Totally agree and agree	25 (22)	*N* = 158
Neutral	24 (15)	
Totally disagree and disagree	99 (62)	

*SD* standard deviation. Numbers do not always add up to 223 due to missing values. Percentages do not always add up due to rounding

^a^The total number of type of work changes is higher compared to the number of change in employment status due to cancer since some participants reported more than one type of work change due to cancer

### Insurance outcomes

**Fig. 1:**
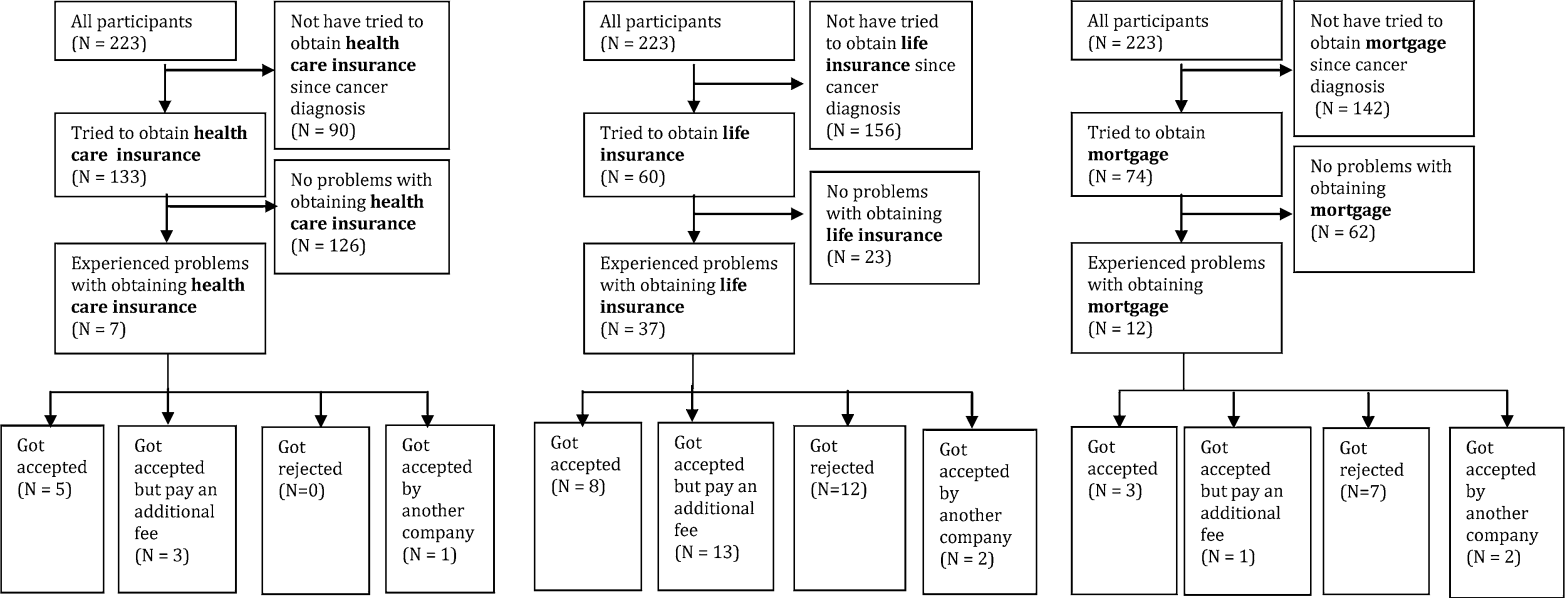
Insurance outcomes among thyroid cancer survivors. Numbers do not always add up because some participants did not fill in the questions correctly (e.g. some participants answered ‘no’ on the question whether he/she experienced problems with obtaining a health care insurance, but did fill in the question what kind of problem he/she experienced)

Of those thyroid cancer survivors who tried to obtain an insurance after their cancer diagnosis, 62 % (*n* = 37) reported obtaining problems with a life insurance, followed by problems with obtaining a mortgage (16 %; *n* = 12) or health care insurance (6 %; *n* = 7) (Fig. 1). Of the 62 % of patients who reported problems with obtaining life insurance, 37 % got accepted but had to pay an additional fee, 34 % got rejected, 23 % got accepted eventually, and 6 % got accepted by another company. Of the 16 % of the patients who reported problems with obtaining mortgage, 54 % got rejected, 23 % got accepted eventually, 15 % got accepted by another company, and 8 % got accepted but pay an additional fee. Of the 6 % of the patients who reported problems with obtaining health care insurance, 56 % got accepted eventually, 33 % got accepted but pay an additional fee, and 11 % got accepted by another company.

### Factors associated with not having employment

Factors associated with not having employment in the univariate logistic regression analysis were: higher age at time of survey [odds ratio (OR) 1.09, (95 % confidence interval (CI) 1.05–1.13), *p* < 0.01], lower educational level [OR 4.67, (95 % CI 2.35–9.29), *p* < 0.01], unfavourable cancer stage [OR 2.23, (95 % CI 1.06–4.72), *p* = 0.04], higher level of fatigue [OR 1.08, (95 % CI 1.03–1.12), *p* = 0.001], higher level of anxiety [OR 1.08, (95 % CI 1.01–1.17), *p* = 0.04], higher level of depression [OR 1.13, (95 % CI 1.03–1.240, *p* = 0.01], and reporting one or more comorbidities [OR 3.99, (95 % CI 2.15–7.42), *p* < 0.01]. Gender, marital status, type of tumour or the treatment were not related to not having employment.

Multivariate logistic regression analysis of model 1, consisting of socio-demographic variables, showed that higher age at time of survey [OR 1.07, (95 % CI 1.03–1.11), *p* < 0.001] and lower educational level were associated with higher chance of not having employment [OR 3.67 (95 % CI 1.78–7.58), *p* < 0.01]. Multivariate logistic regression analysis of model 2, including clinical variables showed that reporting one or more comorbidities was associated with a higher chance of not having employment [OR 3.58 (95 % 1.90–6.71), *p* < 0.01]. Multivariate logistic regression analysis of variables of model 3, including long-term side effect variables, showed that a higher level of fatigue was associated with a higher chance of not having employment [OR 1.07, CI (95 % CI 1.01–1.12), *p* = 0.02] (Table 3).

**Table 3:** Logistic multivariate models of factors associated with not having employment

	Model 1 OR (95 % CI)	Model 2 OR (95 % CI)	Model 3 OR (95 % CI)	Model 1 + 2 + 3 OR (95 % CI)
Block 1: demographic variables				
Age at survey	1.07 (1.03–1.11)**	–	–	1.07 (1.02–1.11)**
Educational level	3.67 (1.78–7.58)**	–	–	3.22 (CI 1.46–7.09)**
Block 2: clinical variables				
Cancer stage	–	2.02 (0.92–4.43)	–	0.99 (0.39–2.51)
Comorbidity	–	3.58 (1.90–6.71)**	–	2.05 (0.99–4.24)
Block 3: long-term side effects variables				
Fatigue (FAS)	–	–	1.07 (1.01–1.12)*	1.07 (CI 1.01–1.14)*
Depression (HADS)	–	–	1.03 (0.89–1.19)	0.93 (0.79–1.10)
Anxiety (HADS)	–	–	0.99 (0.89–1.11)	1.02 (0.91–1.15)

Bold values indicate *p* < 0.05

Employed versus not having employment

Educational level: high (reference) versus medium/low. Cancer stage: 1 and 2 (reference) versus 3 and 4. Comorbidity: no comorbidity (references) versus one or more comorbidities

*OR* odds ratio, *95 % CI* 95 % confidence interval, *FAS* Fatigue Assessment Scale, *HADS* Hospital Anxiety and Depression Scale

* *p* < 0.05; ** *p* < 0.01

In the final multivariate logistic regression model consisting of a combination of model 1, 2 and 3, higher age [OR 1.07, (95 % CI 1.02–1.11), *p* < 0.01], a higher level of fatigue [OR 1.07, CI (95 % CI 1.01–1.14), *p* = 0.02], and a lower educational level [OR 3.22, (95 % CI 1.46–7.09), *p* ≤0.01] remained associated with not having employment (Table 3).

### Quality of life and employment

Employed cancer survivors scored overall better on the thyroid cancer-specific HRQoL scales as well as on global health and overall quality of life (Table 4). Employed thyroid cancer survivors scored significantly better compared to thyroid cancer survivors without employment on neuromuscular problems (20.1. ± 19.5 vs 32.1 ± 25.9, *p* < 0.01), voice problems (7.7 ± 15.2 vs 16.1 ± 24.9, *p* = 0.02), and on overall quality of life (69.4 ± 16.5 vs 61.7 ± 20.4, *p* < 0.01).

**Table 4:** Quality of life outcomes of thyroid cancer survivors with and without paid employment

Health-related characteristics	Employment	Without employment
THYCA-QoL		
Neuromuscular problems (mean ± SD)	20.1 ± 19.5	32.0 ± 25.9**
Voice problems (mean ± SD)	7.6 ± 15.2	16.1 ± 24.9*
Concentration problems (mean ± SD)	16.4 ± 21.5	18.8 ± 24.6
Sympathetic problems (mean ± SD)	16.8 ± 24.4	25.8 ± 3 0.2
Throat problems (mean ± SD)	10.4 ± 14.6	18.2 ± 23.2
Psychological problems (mean ± SD)	13.6 ± 15.1	18.8 ± 20.7
Sensory problems (mean ± SD)	13.3 ± 18.0	19.6 ± 22.4
Overall quality of life (mean ± SD)	69.4 ± 16.5	61.7 ± 20.4**

THYCA-QoL: thyroid-specific health-related quality of life. Higher score means more problems. QOL: overall quality of life on scale 0–100. Higher score means better QOL. Numbers do not always add up to 223 due to missing values

** *p* < 0.01; * *p* < 0.05

## Discussion

The purpose of our study was: (1) to obtain insight into employment and insurance outcomes of thyroid cancer survivors, (2) to examine factors associated with not having employment, and (3) to study thyroid cancer survivors with and without paid employment on quality of life outcomes. Our finding that many thyroid cancer survivors face problems when obtaining a life insurance and that older, fatigued, and lower educated thyroid cancer survivors may be at risk for not being employed, which may both negatively impact cancer survivors’ financial situation and quality of life.

### Strengths and limitations

Strengths of our study include the use of a population-based sample with a high response rate, which implies that our findings can be generalised to thyroid cancer survivors at large. Furthermore, most studies on cancer and work considered the return to work of employed cancer survivors at diagnosis only, excluding unemployed cancer patients, who just might be at a greater risk of adverse outcomes. Our study also had some limitations. First, the cross-sectional design does not allow us to draw conclusions on the causal effect of the association. However, it might be possible that the identified factors might lead to a higher risk of not being employed rather than being a consequence of not being employed. This assumption is based on the notion that the relationship between fatigue as a cause of thyroid cancer treatment has been well established [15] and the nature of the other factors included in our model (e.g. age). This should therefore be studied with a longitudinal design in future studies.

Second, we were not able to include work-related factors such as physical work and type of occupation (e.g. Ref. [8]) that have been found to be associated with employment in other cancer types. For that reason, our model might not be optimal in explaining all factors that are associated with employment among thyroid cancer survivors. Work-related factors should therefore be included in future studies. Finally, we did not collect data on the year in which a thyroid cancer survivor experienced problems with obtaining a health care or life insurance, or a mortgage. In recent years, many changes in the rules and regulations of insurance companies have taken place. These regulations largely influence the extent to which problems are experienced. We are therefore not able to draw conclusions of who are at the highest risk of experiencing problems with obtaining these insurances and mortgage and recommend including this aspect in future studies.

### Interpretation of findings

In contrast to other cancer types, long-term thyroid cancer survivors have comparable employment rates to the general population [24]. An explanation for this finding might be that most thyroid cancer survivors are relatively young and have better long-term physical and psychological outcomes due to less aggressive forms of cancer treatment (no external radiotherapy or chemotherapy). For instance, long-term thyroid cancer survivors (>10 years) do not have elevated levels of fatigue compared to norm values, in contrast to short-term thyroid cancer survivors (<10 years) [15] and to both short-term and long-term survivors of non-Hodgkin’s lymphoma [25].

Thirty-three per cent of the cancer survivors reported work changes due to cancer, which is higher compared to a study among colorectal and haematological cancer survivors (28 % experienced work changes) [26] and lower compared to a study among prostate, endometrial, and haematological cancer survivors [12]. This latter finding could be explained by the fact that we excluded all patients aged ≥65 years, excluding the work change of retirement.

Higher age, lower educational level, and a higher level of fatigue were associated with not having employment irrespective of clinical factors. This finding is consistent with the literature of other cancer types (e.g. [9]). For instance, a Danish study among haematological cancer survivors found that higher age and lower educational level was associated with not returning to work [27]. Another Danish study among breast cancer survivors found that lower educational level was associated with unemployment [28]. In contrast to other cancer types, we did not find that cancer treatment was associated with employment (e.g. [27]). This finding could also be explained by the less aggressive forms of cancer treatment of thyroid cancer survivors.

We found that only a small proportion of the cancer survivors had problems with obtaining a health care insurance or mortgage, which is consistent with a study among both colorectal cancer survivors and haematological cancer survivors [12]. This finding can be explained by the Dutch health insurance system, which forbids by law risk selection for the basic health insurance scheme based on someone’s medical history. In contrast to the study among both colorectal and haematological cancer survivors, we found that much more cancer survivors had problems with obtaining a life insurance (62 vs 20 %) [12]. Additionally, especially for employed thyroid cancer survivors the effect of not being able to obtain a life insurance might be more prominent as the security for their income is more depending on this life insurance compared to unemployed cancer survivors.

Our study showed that employed thyroid cancer survivors report better global health and overall quality of life compared to thyroid cancer survivors without a paid job, which is consistent with the literature [11]. Our finding that thyroid cancer survivors without employment experience more problems on neuromuscular and voice level compared to employed thyroid cancer survivors indicates that a certain level of physical functioning is needed to be able to work. Surprisingly, we did not find differences between employed thyroid cancer survivors and thyroid cancer survivors without employment on concentration problems, while concentration problems have often been reported as a problem for returning to work in other cancer types [29].

### Recommendations for further research and practice

As the employment rate of thyroid cancer survivors is comparable to the general population, interventions to enhance employment for all thyroid cancer survivors seem not needed. However, as in other cancers it is important to reach thyroid cancer survivors who are older, have a lower educational level, and have a higher level of fatigue with an appropriate intervention that will reduce their risk of not having employment. For instance, such an intervention could consist of cognitive behaviour therapy, as it has been proven effective in reducing fatigue among cancer survivors [30]. However, its effect on employment is unknown. Furthermore, health care professionals should be aware of the socio-economic implications of a thyroid cancer diagnosis: in particularly which patients have a higher risk of not having employment and that survivors may face problems with obtaining a life insurance. When health care professionals are aware of these implications, they will be able to point out problems and refer patients to appropriate interventions and authorities.

A recommendation for further research is to include work-related parameters such as type of work or previous unemployment spells in the study. In this way, a model that explains employment would most likely be more complete and could provide further information on which patients are at the highest risk and which parameters should be addressed in an intervention. In addition, on the traditional work outcome, employment rate, thyroid cancer survivors have comparable outcomes compared to the general population. However, as unemployment often is considered the tip of the iceberg of possible adverse work outcomes, it would be very relevant to study subtle work outcomes such as quality of working life, work functioning, and work-home balance in the future.

In addition, a recommendation for further research is to use a longitudinal design to examine whether the earlier identified factors also have a causal relation with employment and to obtain insight in labour market transitions. The first is important for the development of interventions; the latter for a better understanding of employment, as being employed is not a fixed event, but many transitions may occur in the working life course.
